# When Mars Versus Venus is Not a Cliché: Gender Differences in the Neurobiology of Alzheimer’s Disease

**DOI:** 10.3389/fneur.2014.00288

**Published:** 2015-01-12

**Authors:** Katherine Amy Lin, P. Murali Doraiswamy

**Affiliations:** ^1^Division of Translational Neuroscience, Department of Psychiatry, Duke University Medical Center, Durham, NC, USA; ^2^Duke Institute for Brain Sciences, Durham, NC, USA

**Keywords:** sex differences, amyloid, hippocampus, biomarkers, treatment

Alzheimer’s disease (AD) is a global public health threat with the prevalence projected to triple over the next 50 years. While substantial progress has been made in understanding the pathological timeline and developing biomarkers, one area remains relatively neglected: the disproportionate burden that women face. Women not only make up the majority of caregivers but also make up over two-thirds of Alzheimer’s patients (1, 2).

Currently, of the several thousand research studies published in the field of AD, only a tiny fraction are devoted specifically to sex differences. The higher prevalence of AD in women has traditionally been assumed to be due to longer female life expectancy. Differences in educational level, cognitive detection-biases, sex hormones, and genetics have been proposed as other possible causes for the gender imbalance. The literature has remained somewhat conflicting as to whether there is a higher age-adjusted incidence of AD in women with some studies finding no difference (3, 4) and others suggesting that women have a higher incidence (5–8). Perhaps, most striking is epidemiological evidence from the Framingham study, which reported that the age-specific lifetime risk of AD is nearly twofold greater in women than in men – 17.2 versus 9.1% at age 65 years and 28.5 versus 10.2% at age 75 years (6). This has sparked further attention into how gender may influence AD pathophysiology (2).

Cognitive tests commonly used to screen for AD or measure progression in routine practice (e.g., MMSE) are also known to show gender differences – suggesting that factors such as detection bias, cognitive reserve/education, or socio-cultural effects could potentially also contribute to gender differences in AD rates. For example, a meta-analyses of cognitive test scores in AD studies has found that women perform significantly worse than men (9). However, the emergence of biomarker tests, such as CSF, brain volumetric MRI, and amyloid and tau PET scans, has allowed for a more unbiased investigation of how gender affects pathology and neuronal loss at both preclinical and clinical stages of the disease. Selected studies indicative of gender differences in atrophy, pathology, longitudinal cognitive changes, and diagnostic progression in AD are summarized below.

Data from the Alzheimer’s Disease Neuroimaging Initiative (ADNI) have been used to examine gender differences in biomarkers and cognition. Hua et al. examined 1-year atrophy rates, using 3D-tensor based MRI morphometry in 1368 MRI scans (144 subjects with AD, 338 subjects with MCI, and 202 controls scanned twice) and found that annual atrophy rates were faster in women by 1–1.5% (10). Further, atrophy rates correlated with amyloid-beta and tau changes in CSF and with APOE4 allele status. Holland et al. (11) studied 668 subjects (normal, MCI, and AD) and examined gender effects on brain atrophy and cognitive decline (on ADAS-Cog and CDS-SB) over a 3-year-period in a linear mixed effects model controlling for age, education, ApoE4, and baseline cognition. In general, women showed greater atrophy rates and faster cognitive decline than men with the added contribution of female gender being equal to the magnitude of the ApoE4 effect.

In a longitudinal clinicopathologic study of 141 individuals with AD, MCI, or cognitive impairment, using clinical and post-mortem data, a significant correlation was found between gender and neuritic plaques and neurofibrillary tangles after controlling for age (12). In the same investigation, each unit increase on a global measure of AD pathology was found to increase odds of clinical AD by 20-fold for women as opposed to 3-fold for men (12). With each additional unit of global AD pathology, the cognitive function scores in episodic memory, semantic memory, working memory, perceptual speed, and visuospatial ability were reduced significantly more in women than in men (12), suggesting a greater cognitive vulnerability to AD pathology in women, or greater cognitive reserve in men. In a separate study, steeper rates of cognitive decline, decreasing brain volume, and progression from MCI to AD, have been found in women (13).

Studies have also examined whether genetic and hormonal mechanisms contribute to the sex disparities in Alzheimer’s risk and pathology. Some have indicated a greater potency of the risk associated with APOE4 allele in women (14). Changes in fMRI default mode connectivity and increased CSF tau level in APOE4 carriers have been linked to female gender (15). Premature centromere separation (PCS), a consequence of chromosome instability, has been shown to be more common in both females with AD and normal females than in men (16), and in AD, the X-PCS phenotype is accelerated in women (17). Carriers of X-linked mutations show progressive neurodegeneration and ataxia with age (17); together with the finding of X-PCS predominance in AD, this suggests a susceptibility of the X-chromosome to AD-driven changes. Significantly, the AD brain demonstrates a twofold increase of X-chromosome aneuploidy rates in neural cells of the hippocampus and cerebrum, which are the brain areas most affected by neurodegeneration (18). At the epigenetic level, X-inactivation patterns affecting both coding and non-coding regions may cause a female individual to face both a large gene dosage and sex-specific effects (19), which could disproportionately increase female vulnerability to AD. Mean X-chromosome expression has also shown to be associated with neuronal density (20).

Estrogen has been shown to potentially reduce amyloid-beta aggregation and improve a variety of neural functions (hippocampal dendritic spine health, cerebral blood flow and glucose metabolism, increase choline acetyltransferase activity, etc.) (13). Hence, the sharp decline in estrogen levels during menopause could be a significant contributor. Four estrogen receptor beta (ESR2) single-nucleotide polymorphisms (SNPs) were found to be associated with increased risk of AD (21). Sex hormones, including estrogen, may also be involved in promoting non-amyloidogenic pathways (alpha-secretase pathway) and decreasing amyloid-beta production (22). However, in the 5-year WHIMS study, risk of MCI or AD diagnosis increased by 37% in an estrogen plus progestin treatment cohort (23) and deleterious effects on frontal lobe and hippocampal volumes were observed in women assigned to hormone therapy (24). Estrogen and testosterone replacement therapies have also not benefited AD patients in multicenter controlled trials. Reconciling these paradoxical findings, given all the nuances of hormonal therapy, should be priority for the field.

Our current understanding of AD is that its pathogenesis may begin decades before the manifestation of clinical dementia – a stage now termed as preclinical AD. Indeed, in people at risk for familial autosomal dominant AD (carriers of PSEN and APP mutations), silent amyloid-beta changes have been noted some 25 years before predicted onset of clinical disease. Available evidence does not pinpoint any one biological basis for sex differences in Alzheimer’s susceptibility but suggest that gender affects multiple processes in AD including the manifestation of genetic risk, cognitive reserve, cognitive testing performance, brain atrophy rates, and neurotransmitter profiles. However, existing biomarker studies on gender differences in AD have been largely *post hoc* and exploratory in nature. Further examination of gender effects in longitudinal multicenter studies, such as Alzheimer Disease Neuroimaging Initiative-2 (ADNI-2), Dominant Inherited Alzheimer Network (DIAN), Alzheimer’s Prevention Initiative (API), the Amyloid Lowering Trial in Asymptomatic Individuals (A4 trial), as well as ongoing large population studies (e.g., Baltimore Study of Aging, Framingham Study, Women’s Health Initiative, Rotterdam Study of Aging) could be next steps. If gender differences are confirmed, then current models of the timeline of biomarker evolution in AD should be modified to incorporate timeline curves specific for men versus women. Indeed, gender-stratified clinical treatment trials may be logical if gender-specific pathological differences exist.

Over the next 50 years, barring a cure, the share of the burden born by women may rise much faster than it will for men. It is time to set aside old stereotypes, and prioritize gender-specific research in AD.

## Conflict of Interest Statement

The authors declare that the research was conducted in the absence of any commercial or financial relationships that could be construed as a potential conflict of interest. P. Murali Doraiswamy has received grants fromand served as an advisor/speaker to several pharmaceutical and health companies in this field. He owns stock in several companies whose products are not discussed here.
